# Association of Loneliness and Social Isolation With Healthspan and Lifespan in the U.S. Health and Retirement Study

**DOI:** 10.1093/geroni/igaa057.538

**Published:** 2020-12-16

**Authors:** Christopher Crowe, Dayoon Kwon, Daniel Belsky

**Affiliations:** 1 Columbia University, New York, New York, United States; 2 Columbia University Mailman School of Public Health, New York, New York, United States; 3 Columbia Mailman School of Public Health, New York, New York, United States

## Abstract

Loneliness and social isolation present emerging public health challenges for aging populations. To inform efforts against loneliness and social isolation, research is needed to evaluate impacts of these conditions on patterns of adverse aging outcomes. We used longitudinal, repeated measures data to isolate effects of loneliness and social isolation from effects of socioeconomic circumstances and psychological vulnerabilities, rule out reverse-causation, and test dose-response in associations with morbidity, disability, and mortality. We also explored the biological basis of relationships by testing associations with biological aging. We analyzed data from more than 12,000 adults aged 50-95 in the US Health and Retirement Study (HRS) who reported on symptoms of loneliness and social isolation at 2-3 time points during 2006-2014. We measured loneliness using the 3-item Revised UCLA Loneliness Scale and social isolation using a 6-item scale. We tested associations with chronic disease, disability, and biological aging in 2016 and with mortality through 2018. We found (1) associations of loneliness and social isolation with adverse aging outcomes are not fully explained by socioeconomic circumstances and psychological vulnerabilities; (2) the direction of relationships with disability indicates temporal precedence of loneliness and social isolation; (3) there is a dose-response in associations with loneliness; and (4) there is evidence of a biological mechanism explaining associations with loneliness and social isolation. Findings add to the emerging literature on health sequelae of loneliness and social isolation. Assessment of loneliness and social isolation in surveillance and intervention with older adults can enhance risk assessment for adverse aging outcomes.

